# Safety of early oral ambulatory treatment of adult patients with bloodstream infections discharged from the emergency department

**DOI:** 10.1128/aac.00780-23

**Published:** 2023-10-27

**Authors:** Ana Casado, Adelina Gimeno, Manuela Aguilar-Guisado, Manuel García, Jesús Francisco Rodríguez, Pedro Antonio Rivas, Claudio Bueno, Jose Antonio Lepe, José Miguel Cisneros, José Molina

**Affiliations:** 1 Unit of Infectious Diseases, Microbiology and Parasitology, Virgen del Rocío University Hospital, Seville, Spain; 2 Institute of Biomedicine of Seville (IBiS), Virgen del Rocío University Hospital/CSIC/University of Seville, Seville, Spain; 3 CIBER de Enfermedades Infecciosas, Instituto de Salud Carlos III, Madrid, Spain; 4 Emergency Department, Virgen del Rocío University Hospital, Seville, Spain; Johns Hopkins University School of Medicine, Baltimore, Maryland, USA

**Keywords:** Oral step-down antibiotic therapy, Intravenous-to-oral antibiotic switch, antimicrobial stewardship, bloodstream infections, occult bacteremia, emergency department

## Abstract

This study evaluates the safety of early oral ambulatory treatment of adult patients diagnosed with bacteremia after their discharge from the emergency department. A cohort of 206 febrile ambulatory patients was assessed. Bacteremic low-risk patients were recommended an oral treatment and were compared with matched febrile non-bacteremic outpatients. Rates of 14-day mortality and unplanned re-consultations were similar and below 5% in both cohorts, highlighting the safety of oral therapy of low-risk bacteremia, even from its onset.

## INTRODUCTION

Randomized trials have unanimously shown the benefits of oral antibiotic treatments for stable patients with bacteremia (1). However, transition to oral therapy has been usually performed after several days of intravenous treatment—frequently up to 7 days (2)—highlighting the need for evidence of the safety of earlier oral treatments (3).

Occult bloodstream infections (oBSIs) are defined as those bacteremias diagnosed after the patient is discharged from the emergency department (ED) (4). Despite their low mortality (<5%) (4 – 6), the lack of evidence on the safety of ambulatory treatment has led to hospital admission rates after the oBSI diagnosis between 42% and 82% (4 – 6 ), in order to prompt parenteral treatment.

This study aims to evaluate the safety of the oral ambulatory treatment of patients with oBSI, proposing this syndrome as a proof-of-concept for the feasibility of oral treatment of bacteremic infections even from its early onset.

### Design

Retrospective observational matched-cohort study.

### Setting

The study was performed at University Hospital Virgen del Rocío (Spain). In our center, the microbiology department reports to the infectious diseases (ID) department all patients with positive blood cultures on a 24/7 basis. Patients with low-risk oBSI are routinely managed as outpatients, according to the criteria in Table 1. These criteria are regularly applied by the ID physician in charge of the ED area during weekdays and have been transmitted through educational sessions to the rest of the ID attendants to homogenize clinical management during on-call duty.

**TABLE 1:** Clinical criteria for patients with low-risk oBSI eligible for outpatient management

1. Early response to empiric antibiotic treatment: patient states to be asymptomatic or paucisymptomatic at first telephonic contact when the diagnosis of oBSI is informed.
2. Active oral therapy options are available for the microorganism responsible for the oBSI.^*a*^
3. No clinical suspicion of a complicated source for oBSI, potentially requiring source control measures
4. No severe immunosuppression at oBSI diagnosis (current neutropenia, recent transplantation, etc.)
5. Adequate accessibility to ensure close ambulatory follow-up and feasibility to return to the ED if needed.

^*a*^ oBSI produced by *Staphylococcus aureus* and *Candida* sp. are specifically precluded from outpatient management.

### Objective

The objective of this study was to evaluate the safety of early oral ambulatory treatment of patients with oBSI.

### Study population

Adult patients with blood cultures drawn at the ED between March 2020 and November 2021 were consecutively assessed. Two cohorts were followed up: (i) bacteremic febrile patients (BFPs), defined as those discharged from the ED with a febrile syndrome, in whom an oBSI was demonstrated and for whom the ID team recommended an outpatient management; and (ii) non-BFPs, defined as those discharged from the ED with a febrile syndrome, with negative blood cultures. Each BFP was matched with a non-BFP, according to these criteria: (i) first negative blood culture received in chronological order immediately after the BFP blood sample and (ii) the patient should have the same age as the BFP (±10 years). Patients in palliative care and contaminated cultures (defined by a unique positive set of blood cultures for a skin commensal organism) were excluded.

### Endpoint

The primary endpoint was unplanned consultation in the emergency department (UCED) 30 days after the first ED visit. Secondary endpoints were all-cause 14- and 90-day mortality.

### Statistical analysis

Differences between BFP and non-BFP were assessed through univariate analyses. As a sensitivity analysis, multivariate logistic regression was developed pooling both cohorts to determine factors associated with primary and secondary outcomes. Data were obtained through regional healthcare system database consultation.

### Sample size

UCED rates of 17% have been reported for febrile patients discharged from EDs (7). Cohorts comparing bacteremic and non-bacteremic febrile hospitalized patients showed relative risk (RR) of up to 2 for unfavorable outcomes among patients with bacteremia (8 ). No studies were identified assessing outpatients. Using these data and assuming an alpha error of 0.05 and a power of 80%, we calculated a sample size of 206 patients (103 per group).

### Results

During the study period, 193,400 patients were attended at the ED, with a global rate of UCED at 72 hours of 6.4%. Blood cultures were performed in 5,205 patients, and 777 were positive (14.9%).

Overall, 123 patients were diagnosed with oBSI. Outpatient management was recommended to 103 of these patients (83.7%). Ninety-nine of them (96.1%) finished their follow-up without hospitalization. No deaths were reported on day 14 after diagnosis, and the 90-day mortality rate was 1.9%. Clinical features of the 20 patients requiring hospital admission on oBSI diagnosis are detailed in Table S1.

Baseline characteristics and outcomes of BFP and non-BFP are shown in Table 2, and data on specific antibiotic treatments and dosing are detailed in Table 3. Urinary infections predominated among BFP (73.8% vs 38.8%, *P* < 0.001), while non-focal fever was more frequent among non-BFP (8.7% vs 35%, *P* < 0.001), and empirical antibiotic treatment was started at ED discharge less frequently among the latter (92.2% vs 74.8%, *P* = 0.001). BFP and non-BFP presented low and similar rates of 30-day UCED (4.9 vs 3.9%, *P* = 0.73) and 14-day (0 cases) and 90-day (1.9 vs 8.7%, *P* = 0.058) mortality.

**TABLE 2:** Baseline characteristics and outcome differences between BFPs and non-BFPs discharged from the ED and managed as outpatients^*i*^

	BFP (*N* = 103), *n* (%)	Non-BFP (*N* = 103), *n* (%)	*P*
Age (years), median (IQR)	65 (57 to 75)	64 (54 to 75)	0.66
Sex (female)	47 (45.6)	45 (43.7)	0.89
Charlson index score, median (IQR)	5 (3 to 6)	5 (3 to 8)	0.25
Chronic heart failure	9 (8.7)	6 (5.8)	0.42
Diabetes mellitus	21 (20.4)	21 (20.4)	1
Chronic kidney disease	20 (19.4)	17 (16.5)	0.59
Malignancies	37 (35.9)	46 (44.7)	0.26
Acquisition of the suspected infection			
Community	60 (58.3)	62 (60.2)	0.89
Healthcare-related	43 (41.7)	41 (39.8)	0.89
Pitt score at first ED visit, median (IQR)	0 (0–0)	0 (0–0)	1
Definite source for the febrile syndrome			
Urinary	76 (73.8)	41 (39.8)	<0.0001^*a*^
Abdominal	10 (9.7)	9 (8.7)	-
Skin/soft tissue	6 (5.8)	6 (5.8)	-
Vascular	4 (3.9)	0 (0)	-
Respiratory	3 (2.9)	11 (10.7)	-
Unknown	4 (3.9)	36 (35.0)	<0.0001^*b*^
Etiology			
Enterobacterales^*c*^	82 (79.6)	–	–
Non-fermenting GNB^*d*^	10 (9.7)	–	**–**
*Streptococcus* spp.^*e*^	7 (6.8)	–	–
*Staphylococcus* spp. (CoNS)	3 (2.9)	–	–
*Enterococcus faecalis*	1 (0.9)		
Other^*f*^	2 (2.9)	–	–
Empirical antimicrobial therapy started at ED discharge	95 (92.2)	77 (74.8)	0.001
Inappropriate empirical treatment^*g*^	9 (8.8)	5 (5.3)	0.41
Unplanned consultation at ED (30 days)	5 (4.9)	4 (3.9)	1
Hospital admission (30 days)	4 (3.9)	3 (2.9)	1
14-day mortality	0 (0)	0 (0)	1
90-day mortality	2 (1.9)^*h*^	9 (8.7)	0.058

^*a*^ The reference category for this analysis was “non-urinary source.”

^*b*^ The reference category for this analysis was “non-unknown source.”

^*c*^ Enterobacterales*: Escherichia coli* (54), *Klebsiella pneumoniae* (11), *Klebsiella oxytoca* (4), *Proteus mirabillis* (3), *Enterobacter cloacae* (3), *Klebsiella aerogenes* (2), *Citrobacter freundii* (1), *Citrobacter braakii* (1), *Pantoea agglomerans* (1), *Proteus vulgaris* (1), and *Aeromonas veronii* (1). Two of the *K. pneumoniae* bacteremias were polymicrobial: one was mixed with *E. coli* and another with *E. faecalis*.

^*d*^ Non-fermenting GNB: *Pseudomonas aeruginosa* (5), *Achromobacter xylosoxidans* (1), *Acinetobacter baumannii* (1), *Acinetobacter piti* (1), *Moraxella osloensis* (1), and *Roseomonas mucosa* (1).

^*e*^ *Streptococcus* spp.: *Streptococcus pneumoniae* (3), *Streptococcus agalactiae* (2), *Streptococcus anginosus* (1), and *Streptococcus dysgalactiae* (1).

^*f*^ Other: *Parabacteroides distansonis* (1) and *Aerococcus viridans* (1).

^*g*^ Inappropriate empirical treatment was defined based on lack of adherence to local antibiotic policy, according to the suspected diagnosis at first ED visit (9).

^*h*^ Deceased patients in the BFP group: one patient with acute myeloid leukemia died 46 days after the first ED visit due to an unrelated cause (invasive aspergillosis); one patient with metastatic pancreatic cancer died 81 days after the first ED visit due to an unrelated cause (tumoral progression).

^*i*^ BFP, bacteremic febrile patient; CoNS, coagulase-negative *Staphylococcus*; ED, emergency department; GNB, Gram-negative bacillus; IQR, interquartile range.

**TABLE 3:** Antimi­crobial agents and dosing^*a*^

	BFP (*N* = 103), *n* (%)	Non-BFP (*N* = 103) *n* (%)
Patients receiving any dose of empirical parenteral antibiotics before ED discharge^*b*^	28 (27.2)	21 (20.4)
Parenteral antibiotic agents and dosing		
Ceftriaxone 1 g/24 hours	13 (46.4)	10 (47.6)
Ceftriaxone 2 g/24 hours	6 (21.4)	4 (19.0)
Piperacillin-tazobactam 4 g/500 mg/8 hours	3 (10.7)	2 (9.5)
Amoxicillin-clavulanic acid 1 g/200 mg/8 hours	2 (7.1)	4 (19.0)
Other	4 (14.3)	1 (4.8)
Number of parenteral doses administered before ED discharge, median (IQR)	1 (1 to 1)	1 (1 to 1)
Empirical oral antibiotics started at ED discharge	95 (92.2)	77 (74.8)
Penicillin	16 (16.8)	25 (32.5)
Amoxicillin	0 (0)	2 (2.6)
500 mg/8 hours	0	2
Amoxicillin-clavulanic acid	16 (15.5)	23 (29.9)
500 mg/125 mg/24 hours^*c*^	0	1
500 mg/125 mg/8 hours	0	2
875 mg/125 mg/8 hours	16	20
Cephalosporin	63 (66.3)	35 (45.4)
Cefadroxil	3 (2.9)	2 (2.6)
500 mg/12 hours	0	1
500 mg/8 hours	1	1
1,000 mg/12 hours	1	0
1,000 mg/8 hours	1	0
Cefuroxime	0 (0)	2 (2.6)
500 mg/24 hours	0	2
Cefixime	59 (62.1)	31 (40.2)
200 mg/24 hours^*c*^	0	2
400 mg/24 hours	59	28
400 mg/12 hours	0	1
Cefditoren	1 (1.0)	0
400 mg/24 hours	1	0
Fluorquinolone	11 (11.6)	12 (15.6)
Ciprofloxacin	7 (7.4)	7 (9.1)
500 mg/24 hours	1	0
500 mg/12 hours	5	6
750 mg/12 hours	1	1
Levofloxacin	4 (4.2)	5 (6.5)
250 mg/24 hours^*c*^	1	0
500 mg/24 hours	3	3
750 mg/24 hours	0	1
500 mg/12 hours	0	1
Other	5 (5.2)	5 (6.5)
Definite oral antibiotics after BSI diagnosis	103 (100)	–
Penicillin	14 (13.6)	–
Amoxicillin	4 (3.9)	–
1000 mg/8 hours	4	–
Amoxicillin/clavulanic acid	10 (9.7)	–
500/125 mg/12 hours^*c*^	1	–
875/125 mg/8 hours	9	–
Cephalosporin	63 (61.2)	–
Cefadroxil	2 (1.9)	–
500 mg/8 hours	1	–
1,000 mg/8 hours	1	–
Cefixime	61 (59.2)	–
200 mg/24 hours^*c*^	1	–
400 mg/24 hours	60	–
Fluorquinolone	21 (20.4)	–
Ciprofloxacin	14 (13.6)	–
500 mg/24 hours^*c*^	1	–
500 mg/12 hours	6	–
750 mg/12 hours	7	–
Levofloxacin	7 (6.8)	–
250 mg/24 hours^*c*^	2	–
500 mg/24 hours	3	–
750 mg/24 hours	2	–
Other	5 (4.8)	–
Total treatment duration, median (IQR)	10 (8 to 14)	7 (7 to 10)

^*a*^ ED, emergency department; IQR, interquartile range.

^*b*^ These patients were prescribed at least one dose of empirical parenteral antibiotics according to the ED physician’s clinical judgment during the first attention of the febrile syndrome, before knowing the diagnosis of bacteremia and before the ED discharge.

^*c*^ These patients received dose adjustment due to chronic renal failure.

Multivariate analyses were performed for detecting factors independently related to unfavorable outcomes (Table S2). No variables were associated with increased UCED or 14-day mortality, including belonging to the cohort of bacteremic patients treated orally, the selection of any specific antibiotic class as definite therapy, or the use of an initial intravenous dose of antibiotics during the initial ED attendance. Malignancies (RR 6.69, 95% CI 1.39–32.18) and Pitt score at first ED visit (RR 8.69, 95% CI 2.16–34.96) were associated with 90-day mortality.

### Discussion

Our study supports the safety of the oral, ambulatory treatment of patients with low-risk oBSI, whose outcomes were similar to other febrile patients without bacteremia routinely attended as outpatients. To our knowledge, this is the first comparative study assessing the safety of this approach for bacteremic infections.

Randomized trials have unanimously shown the benefits of switching to oral treatments in stable patients with bacteremia (1), but the duration of previous intravenous treatment varies widely across trials. Vandenbroucke et al. (10) found no effect on oral bioavailability of antibiotics during the acute phase of infection in non-critically ill patients. While there seems to be no good reasons for switching to intravenous therapy for low-risk bacteremic patients rapidly responding to oral treatment, admitting these patients for parenteral treatment after the diagnosis of oBSI has been the standard of care in many centers (4, 5). Our results may challenge the current state of the art, suggesting that no minimum duration of intravenous lead-in is required in all patients with bacteremia and that switching to oral therapy can be performed as soon as clinical criteria are met. In fact, in our sample, the majority of bacteremic patients received no doses of intravenous antimicrobials at all. This strategy would be efficient and equally safe in the case of low-risk BSI, considering that 96.1% of these patients could avoid hospitalization. Our sample fairly represents patients with oBSI, since 103 of 123 patients could be treated orally, a datum that highlights the feasibility of this approach. However, an accurate assessment of patients’ risk by ID experts would be essential to ensure its safety. Noticeably, we recorded no deaths during the first 14 days after oBSI diagnosis, even among those few patients requiring an unplanned admission.

Another remarkable aspect of our results is that most BFPs were managed with oral betalactams with good outcomes. Most studies performed to date advocate for quinolones as the preferential treatment for intravenous-to-oral switch due to their high bioavailability (1). Increased recurrence rates of bacteremia have been observed in some studies when betalactams were used (11 ), but this may be due to insufficient dosing of oral betalactams, as suggested by later works (12, 13 ). In our center, high-dose oral betalactams are recommended as first-line empirical treatment due to quinolone resistance (Table 3) (14 ). Our results suggest that high-dose oral betalactams may be a good alternative for low-risk bacteremic patients, but our sample was not powered to assess differences between agents.

Our study has some limitations. First, despite performing sample size calculations, the cohort may be underpowered to detect small differences. However, the low incidence of complications in absolute terms supports our hypothesis. Second, there were imbalances between groups regarding a higher rate of non-focal fever and lower rates of empirical treatments among non-BFP. These differences probably respond to the requirement of negative blood cultures for non-BFP, which may have favored the inclusion of non-bacterial fever in this group. Clinicians may have been reluctant to initiate empirical treatment for these cases. In our opinion, these differences define a profile of less-severe patients in the non-BFP cohort, which would reinforce our hypothesis.

In conclusion, the early oral, ambulatory treatment of low-risk bacteremic infections seems feasible and safe. These results provide a proof of concept for the efficacy of oral antibiotic therapy for bacteremia even from its onset and may enable outpatient care programs for these infections.

## SUPPLEMENTAL MATERIAL

Supplemental file 1 aac.00780-23-s0001.docx Tables S1 to S3

ASM does not own the copyrights to Supplemental Material that may be linked to, or accessed through, an article. The authors have granted ASM a non-exclusive, world-wide license to publish the Supplemental Material files. Please contact the corresponding author directly for reuse.
