# Supplementary material for: Safety of early oral ambulatory treatment of adult patients with bloodstream infections discharged from the emergency department
Source: Antimicrob Agents Chemother. 2023 Oct 27;67(11):e00780-23. doi: 10.1128/aac.00780-23 (PMC10648851; doi:10.1128/aac.00780-23)
Supplement: Supplemental file 1 — Tables S1 to S3 [file aac.00780-23-s0001.docx]

**Supplementary tables.**

**Supplementary table 1. Clinical features of patients with a diagnosis of occult bloodstream infection which were not eligible for ambulatory management.**

|  | **N=20**  **n (%)** |
| --- | --- |
| **Age (years)** (median, IQR)) | 71 (64-79) |
| **Charlson index score** (median, IQR) | 6.5 (5-10.5) |
| **Definite source for the febrile syndrome** |  |
| Urinary | 7 (35) |
| Abdominal | 7 (35) |
| Skin/soft tissue | 2 (10) |
| Endovascular | 3 (15) |
| Unknown | 1 (5) |
| **Etiology** |  |
| *Enterobacterales* | 8 (40) |
| Non-fermenting GNB^2^ | 1 (5) |
| *Streptococcus* spp. | 2 (10) |
| *Staphylococcus aureus* | 1 (5) |
| *Staphylococcus epidermidis* | 1 (5) |
| *Enterococcus* spp*.* | 3 (15) |
| Anaerobes | 2 (10) |
| Other | 2 (10) |
| **Hospital admission** | 20 (100) |
| **Reasons for ineligibility for ambulatory management detected at the diagnosis of bacteremia** |  |
| Persisting or worsening symptoms at diagnosis | 12 (60) |
| A complicated source was suspected | 4 (20) |
| No oral treatment options were available | 3 (15) |
| Severe immunosuppression | 1 (5) |
| **14-day mortality** | 0 (0) |
| **30-day mortality** | 1 (5) |
| **90-day mortality** | 4 (20) |

**Supplementary table 2. Variables independently associated with primary and secondary endpoints.**

1. ***Analysis of variables related to unplanned consultations at the Emergency Department (UCED) 30 days after the first visit to the ED.***

| **Variable** | **UCED** | **Unadjusted** | | **Adjusted** | |
| --- | --- | --- | --- | --- | --- |
|  |  | ***p*** | **RR (CI95%)** | ***p*** | **RR (CI95%)** |
| **Age (mean±SD)**  *(UCED vs. non-UCED)* | 66.83±14.71 *vs.* 64.28±15.66 | 0.66 | 1.01 (0.96 - 1.06) | - | - |
| **Charlson (mean±SD)**  *(UCED vs. non-UCED)* | 4.33±2.91 *vs.* 5.22±3.55 | 0.46 | 0.92 (0.75 - 1.14) | 0.58 | 0.94 (0.74-1.16) |
| **Diabetes Mellitus** *(yes vs. no)* | 0/42 (0) *vs.*  9/164 (5.5) | 0.2 | - | - | - |
| **Chronic kidney disease**  *(yes vs. no)* | 2/37 (5.4) *vs.*  7/169 (4.1) | 0.67 | 1.32  (0.26-6.64) | - | - |
| **Malignancy**  *(yes vs. no)* | 3/83 (3.6) *vs.* 6/123 (4.9) | 0.74 | 0.73 (0.18-3) | - | - |
| **Community acquisition**  *(yes vs. no)* | 5/122 (4.1) *vs.* 4/84 (4.8) | 0.82 | 1.17 (0.35 - 4.49) | - | - |
| **Year**  *(2020 vs. 2021)* | 0/52 (0) *vs.*  9/154 (5.8) | 0.12 | - | - | - |
| **Definitive urine source** *(yes vs. no)* | 4/116 (3.4) *vs.* 5/90 (5.6) | 0.51 | 0.60 (0.16-2.33) | 0.34 | 0.46 (0.09-2.24) |
| **Pitt score (mean±SD)**  *(UCED vs. non-UCED)* | 0.11±0.33 *vs.* 0.09±0.32 | 0.81 | 1.25 (0.19 - 8.2) | - | - |
| **Empirical antimicrobial therapy** *(yes vs. no)* | 9/172 (5.2) *vs.* 2/34 (5.9) | 1 | 0.88 (0.18-4.28) | - | - |
| **Inappropriate empiric treatment** *(yes vs. no)* | 1/14 (7.1) *vs.* 7/182 (3.8) | 0.45 | 1.93 (0.22-16.83) | 0.78 | 1.36 (0.13-13.45) |
| **Definite quinolone therapy** *(yes vs. no)* | 1/33 (3.0) *vs.* 9/147 (6.1) | 0.69 | 0.48 (0.59- 3.92) | 0.59 | 1.83 (0.20- 16.50) |
| **Definite cephalosporin therapy***  *(yes vs. no)* | 4/98 (4.1) *vs.*  6/82 (7.3) | 0.51 | 0.53 (0.15-1.98) |  |  |
| **Definite penicillin therapy****  *(yes vs. no)* | 4/39 (10.3) *vs.* 6/141 (4.3) | 0.23 | 2.57 (0.69-9.61) |  |  |
| **Any dose of parenteral therapy before ED discharge*****  *(yes vs. no)* | 2/49 (4.1) *vs.* 9/157 (5.7) | 1 | 0.70 (0.14-3.35) | - | - |
| **BFP *vs.* nonBFP** | 5/103 (4.9) *vs.* 4/103 (3.9) | 1 | 1.26 (0.32-4.84) | 0.65 | 1.49 (0.31-7.23) |

**RR**: Relative Risk; **CI95%**: Confidence Interval 95%; **SD:** Standard deviation; **UCED:** unplanned consultations at the Emergency Department; **BFP**: Bacteremic febrile patients; **nonBFP**: non-bacteremic febrile patients.

***Definite cephalosporin therapy** included patients treated with oral 2^nd^ o¡and 3^rd^ generation cephalosporins as definite therapy after blood culture results.

****Definite penicillin therapy** included patients treat with oral amoxicillin or amoxicillin-clavulanate as definite therapy after blood culture results.

***** Any dose of parenteral therapy before ED discharge**: these patients were prescribed at least one dose of empirical parenteral antibiotics according to the ED physician’s clinical judgment during the first attention of the febrile syndrome before knowing the diagnosis of bacteremia, and before the ED discharge.

1. ***Analysis of variables related to 14-day crude mortality.***

No cases of 14-day mortality were recorded in any group.

***(c) Analysis of variables related to 90-day crude mortality.***

| **Variable** | **90-day mortality** | **Unadjusted** | | **Adjusted** | |
| --- | --- | --- | --- | --- | --- |
|  |  | ***p*** | **RR (CI95%)** | ***p*** | **RR (CI95%)** |
| **Age (mean±SD)**  *(dead vs. alive)* | 64.90±19.70 *vs.* 64.36±15.40 | 0.91 | 1.002 (0.96 - 1.04) | - | - |
| **Charlson (mean±SD)**  *(dead vs. alive)* | **8.18±4.55 vs. 5.02±3.39** | **0.006** | **1.24 (1.06 - 1.45)** | 0.30 | 1.09 (0.91-1.31) |
| **Diabetes Mellitus** *(yes vs. no)* | 1/42 (2.4) vs. 10/164 (6.1) | 0.47 | 0.37 (0.05-3.02) | - | - |
| **Chronic kidney disease** *(yes vs. no)* | 3/37 (8.1) vs. 8/169 (4.7) | 0.42 | 1.78 (0.45-7.04) | - | - |
| **Malignancy**  *(yes vs. no)* | **9/83(10.8) vs. 2/123(1.6)** | **0.008** | **7.35 (1.54-34.98)** | **0.008** | **6.69 (1.39-32.18)** |
| **Community- acquired**  *(yes vs. no)* | 7/122 (5.7) vs. 4/84 (4.8) | 1 | 1.21 (0.34-4.29) | - | - |
| **Year**  *(2020 vs. 2021)* | 1/52 (1.9) vs. 10/154 (6.5) | 0.29 | 0.28 (0.03-2.26) | - | - |
| **Definitive urine source**  *(yes vs. no)* | 3/116 (2.6) vs. 8/90(8.9) | 0.06 | 0.27 (0.7-1.1) | 0.53 | 0.62  (0.13-0.75) |
| **Pitt score (mean±SD)**  *(dead vs. alive)* | **0.36± 0.70 vs. 0.07±0.28** | **0.01** | **4.32 (1.4 - 13.35)** | **0.002** | **8.69 (2.16 - 34.96)** |
| **Empirical antimicrobial therapy** *(yes vs. no)* | 10/172 (5.8) *vs.* 1/34 (2.9) | 0.69 | 2.19 (0.27 - 17.64) |  |  |
| **Inappropriate empirical treatment** *(yes vs. no)* | 1/14 (7.1) vs. 9/182 (4.9) | 0.53 | 1.48 (0.17-12.58) | - | - |
| **BFP *vs.* nonBFP** | **2/103 (1.9) vs. 9/103 (8.7)** | **0.047** | **0.21 (0.04-0.99)** | 0.06 | 0.21  (0.04-1.10) |

**RR**: Relative Risk; **CI95%**: Confidence Interval 95%; **SD:** Standard deviation; **BFP**: Bacteremic febrile patients; **nonBFP**: non-bacteremic febrile patients.

Significant differences are highlighted with bold figures.

**Supplementary table 3. Clinical management of patients diagnosed with occult bloodstream infections after the microbiological diagnosis.**

|  | **N=123**  **n (%)** |
| --- | --- |
| **Antibiotic therapy was started by the ID physician in patients not receiving antibiotics at the moment of the bacteremia diagnosis** | 9 (7.3) |
| **Empirical antibiotic therapy was modified by the ID physician after blood cultures results** | 43 (35) |
| **Clinical diagnosis was modified by the ID physician after blood cultures results** | 20 (16.3) |
| **Hospital admission recommended at occult bacteremia diagnosis.** | 20 (16.3) |
